# *Mycobacterium tuberculosis* Infection Is Associated with the Development of Erythema Nodosum and Nodular Vasculitis

**DOI:** 10.1371/journal.pone.0062653

**Published:** 2013-05-01

**Authors:** Sheng’an Chen, Jiazhen Chen, Lianjun Chen, Qiao’an Zhang, Xiaoqun Luo, Wenhong Zhang

**Affiliations:** 1 Department of Dermatology, Huashan Hospital, Fudan University, Shanghai, China; 2 Department of Infectious Diseases, Huashan Hospital, Fudan University, Shanghai, China; Institut de Pharmacologie et de Biologie Structurale, France

## Abstract

**Background:**

*Mycobacterium tuberculosis* (MTB) infection has been suggested to contribute to the pathogenesis of erythema nodosum (EN) and nodular vasculitis (NV), the classic forms of panniculitis. However, there is little evidence to demonstrate the presence of MTB in the skin lesions. This study is aimed at evaluating the association between MTB infection and the development of EN and NV in a Chinese population.

**Methods:**

A total of 107 patients (36 EN, 27 NV, and 44 others) with vasculitis and 40 control cases with other skin diseases were recruited and their skin lesion samples were subjected to real time polymerase chain reaction (PCR) analysis of the IS6110 and *mpt64* gene fragments of MTB. Their blood mononuclear cells were tested for MTB antigen-specific IFN-γ responses by QuantiFERON®-TB Gold In-Tube (IT) assays.

**Results:**

PCR analysis revealed that 7/23 (30.4%) and 7/18 (38.9%) of the EN and NV samples were positive for the IS6110 DNA, respectively, which were significantly higher than 3/34 (8.8%) of other vasculitis (OV) and 3/40 (7.5%) of the control samples (p<0.05). The nested Real-Time PCR assay indicated that 6/7 (86%) of the IS6110-positive EN samples, all of the IS6110-positive NV and control samples, but only 1/3 of the IS6110-positive OV samples, were positive for the *mpt64* gene. Similarly, 19/32 (59.4%) of the EN patients, 20/26 (76.9%) of the NV patients, and 17/36 (47.2%) of the OV patients were positive for MTB antigen-specific IFN-γ responses, which were significantly higher than 6/40 (15%) of the controls (p<0.05).

**Conclusion:**

Our data strongly suggest that MTB infection and active TB are associated with the development of NV and EN in Chinese.

## Introduction

Erythema nodosum (EN) is a frequent pathologic variant of panniculitis in the clinic. The disorder is clinically characterized by the sudden eruption of erythematous tender nodules and plaques located predominantly over the extensor aspects of the lower extremities. EN shows histopathological diversification, including vasculitis, septum interlobular inflammation, hemorrhage, varying degrees of acute or chronic panniculitis, and Miescher’s radial granulomas [1], [2]. There are many etiologic factors that may contribute to the development of EN, and they include infection with streptococcus or *Mycobacterium tuberculosis* (MTB), *Mycobacterium leprae* [3]–[5], severe inflammation, drug-related hyperreactivity, estrogen and malignant diseases [6], [7]. Streptococcal infection is the most common etiological factor for the development of EN, especially in children. Leprosy, drug-related hyperreactivity, hormonal reactions, inflammatory bowel disease, and sarcoidosis are common causative factors of EN in adults. Although MTB infection-related EN is not common [7], there have been some cases reported [8].

Nodular vasculitis (NV) is one type of erythema induratum (EI) and is characterized by recurrent crops of tender oedematous nodules on the calf of the lower legs [8]. NV histologically presents a lobular panniculitis with granulomatous inflammation, vasculitis, focal necrosis, and septal fibrosis [8], [9]. NV has currently been considered as a multifactorial disorder and is associated with bacterial infection, such as streptococcus and MTB, and drug-related hyperreactivity, leading to the vascular inflammation of subcutaneous tissue and lobular panniculitis [7], [10]. However, there is little evidence to demonstrate the etiologic role of MTB in the pathogenesis of EN and NV.

The prevalence of tuberculosis (TB) in advanced countries, such as the United States and Nordic areas, is relatively low, and Streptococcal infection and sarcoidosis are main causative factors of EN and NV [11]. However, TB remains an epidemic in some developing countries and regions, including Thailand, India, Turkey, and the Cape region of South Africa Western, and MTB infection may be the most important factor, contributing to the development of EN and NV [12]–[15]. Notably, the incidence of TB in China is very high [16]–[18]. Approximately, there are 4.5 million patients with active pulmonary TB and about 550 million people with MTB infection. Previous studies have suggested that MTB infection is associated with the development of EN and NV in Chinese patients [19]. However, most of these studies are based on case reports or analysis of retrospective clinical data, and there is little pathologic evidence of MTB in the EN and NV lesions in Chinese patients.

Traditional methods for the diagnosis of active TB include tuberculin skin test (TST), X-ray, biopsy for acid-fast bacilli, and culture for MTB [20]. However, culture of MTB or biopsy for acid-fast bacilli is either time consuming or has low sensitivity. The specificity of TST test is poor for detecting MTB infection. A previous meta-analysis showed that the specificity of TST in BCG-vaccinated populations was low and highly heterogeneous, but the specificity of interferon-gamma (IFN-γ)-release assays was consistent in a BCG-vaccinated population [21]. Our previous studies and those of others have shown that the specificity of TST for detecting MTB infection in China varied from 61.5% to 70.6% [22], [23]. Vaccination of infants with M. bovis BCG is mandatory in China. In addition, the tuberculin used in the TST test contains a crude mixture of more than 200 MTB antigens, which are widely shared with M. bovis BCG and other environmental mycobacteria. It is difficult to use the TST assay to distinguish immune responses from fresh MTB infection and BCG vaccination in the BCG-vaccinated individuals. Hence, we did not use the TST in our present study. In recent years, IFN-γ release assays (IGRAs) has been approved as a new standard for the diagnosis of MTB infection in the clinic. Particularly, QuantiFERON ®-TB Gold in tube can be used for the detection of MTB antigen-specific IFN-γ responses. This assay has a high sensitivity and specificity, and can distinguish MTB infection from BCG vaccination. A previous study has utilized QuantiFERON to detect MTB-specific INF-γ responses in patients with EI [24]. However, whether this assay can be used for the diagnosis of EN and NV has not been clarified.

In this study, we investigated the presence of MTB infection in the skin lesions of 107 patients with suspicious panniculitis and vasculitis by real-time PCR of the IS6110 and *mpt64* gene fragments that are conservative in mycobacterium [9], [25], and we examined MTB-specific INF-γ responses in those patients to explore the importance of MTB infection in the development of EN and NV in Chinese patients.

## Materials and Methods

### Patients and Samples

A total of 226 patients with skin nodules and erythema were recruited at the Department of Dermatology at Huashan Hospital in Shanghai from September, 2010 to November, 2011. Of these, 107 patients were diagnosed as suspicious panniculitis and vasculitis, and classified into the EN, NV, or OV, according to the clinical features and pathological evidence. Patients in the OV group had vascular inflammatory, but not sufficient evidence of EN and NV. Another 40 control patients had clear clinical and pathological manifestations of stasis dermatitis, dermatitis, or eczema. Individual patients were excluded if they had taken anti-MTB drugs, glucocorticoids, and immunosuppressive agents within 3 months. Written informed consent was obtained from individual patients, and the experimental protocol was approved by the Ethics Committee of Huashan Hospital.

Individual patients were subjected to skin biopsy, and their venous blood samples were obtained immediately after diagnosis.

### QuantiFERON^®^-TB Gold in Tube Assay

T cell immunity against MTB in individual patients was determined by QuantiFERON assay using the specific kit, according to the manufacturer’s instruction (Qiagen, Germany). Individual patients were considered as potential MTB infection if the levels of MTB-specific IFN-γ were >0.35 IUmL^−1^ and >25% of the control value. Individual patients were considered as undetermined if the levels of IFN-γ in the control tube were >8.0 IUmL^−1^ or the levels of mitogen-specific IFN-γ were <0.5 IUmL^−1^, and if the levels of MTB-specific IFN-γ were >0.35 IUmL^−1^ and <25% of the control tube. Individuals with IFN-γ level of <0.35 IUmL^−1^ or <25% of the control value were considered as negative for MTB infection.

### Extraction of DNA from Lesion Tissue

The skin tissue samples were cut into small pieces (<2 mm in one dimension) and homogenized in PBS, followed by being digested with 10 mg/ml of lysosome at 37°C overnight. Subsequently, total DNA was extracted from tissue lysates using the QIAamp DNA mini kit (Qiagen, Germany), according to the manufacturer’s instruction, quantified and stored at −20°C.

### Construction of Standard Plasmid

DNA fragments for the *mpt64* (220 bp) and IS6110 (420 bp) genes of MTB were amplified from the MTB H37Rv genomic DNA by PCR using the primers of M64-OUT-F1, M64-OUT-R-link and IS6110-F-link, and IS6110-R1 that were designed and synthesized by TaKaRa Biotechnology (Beijing, China). The sequences of primers are shown in Table 1. The amplifications were performed in triplicate in 50 µl of reactions containing 10 mM dNTP, 3 mM MgCl_2,_ 1×Ex Taq buffer, 0.25 µl of Ex Taq enzyme (Takara), 0.6 µM each of primers, and 1 µl of MTB H37Rv genomic DNA (GenBank accession no. NC_000962) at 95°C for 5 min and subjected to 30 cycles of 94°C for 1 min, 58°C for 1 min, and 72°C for 1 min, followed by extension at 72°C for 5 min. The *mpt64* and IS6110 gene PCR products were purified, linked together, cloned into plasmid of the pUC18 T-vector (Takara, Japan) to generate a new plasmid of pUC18::mpt64+IS6110, and then transformed into *E. coli* DH5α. The recombinant plasmid containing the *mpt64*/IS6110 fragment was extracted using Qiagen Plasmid Kit (Qiagen), quantified, and used as the standard control plasmid for quantitative Real-Time PCR.

**Table 1:** The nucleotide sequences of the primers and probes used in fusion PCR and Real-Time PCR.

Primer names	Sequence (5–3′)	Amplificants size (bp)	Resources
M64-OUT-F1	ATCCGCTGCCAGTCGTCTTCC	239	[27]
M64-OUT-R1	CTCGCGAGTCTAGGCCAGCAT		[27]
M64-OUT-R-link	GAATTCCGATATCTCGCGAGTCTAGGCCAG		This study
IS6110-F1	TTCAGGTCGAGTACGCCTTC	438	[26]
IS6110-R1	CGAACTCAAGGAGCACATCA		[26]
IS6110-F-link	ATATCGGAATTCTTCAGGTCGAGTACGCC		This study
QM3-IS6110-F1	AGGCGAACCCTGCCCAG	122	[29]
QM4-IS6110-R1	GATCGCTGATCCGGCCA		[29]
IS6110_FAM	5′FAM-TGTGGGTAGCAGACCTCACCTATGTGTCGA-TAMRA3′		[29]
M64-Tqm-F2	GTGAACTGAGCAAGCAGACCG	77	[27]
M64-Tqm-R2	GTTCTGATAATTCACCGGGTCC		[27]
MPT64-W-JOE	5′-JOE-TATCGATAGCGCCGAATGCCGG-BHQ1-3′		[27]
HBB-F: human β-globin forward	GGCAGACTTCTCCTCAGGAGTC	196	[27]
HBB-R: human β-globin forward	CTTAGACCTCACCCTGTGGAGC		[27]

### Real-Time PCR (Taqman) Detection and Quantification of the *mpt64* and IS6110 Genes in Tissue DNA

The contents of MTB DNA in individual skin samples were determined by quantitative Real-Time PCR analysis of the *mpt64* and IS6110 genes using the specific primers of QM3-IS6110-F1 and QM4-IS6110-R1 and the probe of IS6110-FAM (Table 1). The amplifications were performed in 18 µl of PCR reactions containing 10 µl of 2×TaqMan PCR master mix (Takara), 0.6 µM each of primers, and 0.4 µM TaqMan probe, 2 µl of tissue DNA or standard plasmid at 50°C for 2 min, 95°C for 15 min, and subjected to 40 cycles of 95°C for 15 sec and 60°C for 1 min.

The contents of the *mpt64* gene in individual skin samples were detected using a two-step nested Real-Time PCR (Taqman probe). In the first round, PCR was performed with the primers of M64-OUT-F1 and M64-OUT-R1. In the second round, Real-Time PCR was performed with Premix Ex Taq ™ reagent, primers of M64-Tqm-F2 and M64-Tqm-R2, and the probe of MPT64-W-JOE. The first round PCR were performed in a final volume of 20 µl containing 100 mM dNTP; 3 mM MgCl2; 1*Ex Taq buffer; 0.25 µl of Ex Taq enzyme (Takara), 0.6 µM each of each primer, and 2 µl of DNA template or standard plasmid. The second round was Real-Time PCR, which had the same condition as of the IS6110.

A series of 10-fold diluted pUC18::mpt64+IS6110 plasmid, from 1×10^9^ copy/ml to 1000 copy/ml, were used for the establishment of a standard curve in Real-Time PCR, and dH_2_O was used as a negative control in each test.

For Real-Time PCR analysis of the IS6110 gene, the fluorescence for detection in Real-Time PCR was FAM, quenching agent was TAMRA, and reference fluorescence was ROX. The fluorescence for detection of *mpt64* was JOE, and the reference fluorescence was ROX. The experiment was considered valid when the R value of the standard curve >0.97. Analyzing with the preset Ct threshold and method of absolute quantitative, the Ct value was converted to copies/µl.

In addition, the human β-globin gene was amplified and used as an internal PCR control using the primers of HBB-F and HBB-R (Table 1).

### Statistical Analysis

Data are expressed as the mean ± SD, median (range), or the real case number and percentage, and all experiments were repeated at least 3 times. The difference among the different groups of patients was analyzed using chi-square, Fisher’s exact test, one-way ANOVA test, or Newman-Kruel non-parametric tests, when applicable. The results from QuantiFERON and real-time PCR assays were compared by McNemar’s test for concordance in each group and all groups of patients who had been simultaneously tested. The association between these two assays was analyzed by Kappa test, and the percentage of agreement was calculated by dividing the numbers of agreement results by all those tested. The difference of % agreements between groups was analyzed by Chi-square test. All statistical analyses were performed using Prism 5 software. A p value of <0.05 was considered statistically significant.

## Results

### Characterization of Groups and Enrolled Subjects

**Figure 1:**
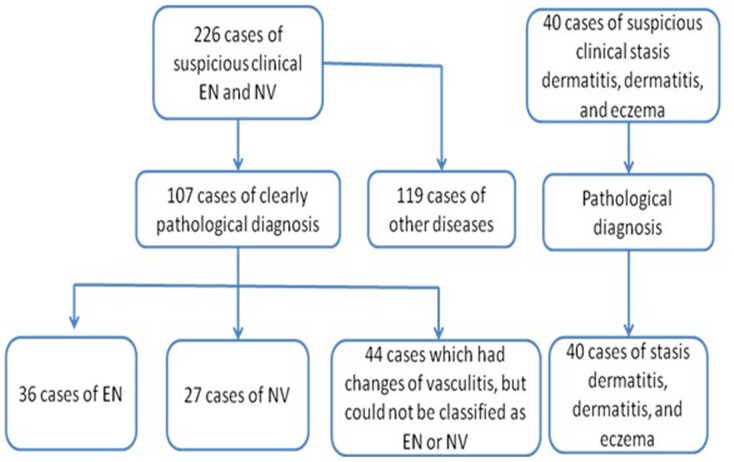
Flowchart of grouping subjects.

A total of 226 patients with suspicious vasculitis were recruited, and 107 patients were pathologically diagnosed with vasculitis. There were 36 cases with EN, 27 cases with NV, and 44 cases with OV, which had characteristics of vasculitis, but could not be classified into EN or NV. The remaining 119 cases were excluded because they suffered from other diseases, including pretibial myxedema, spores of fungus disease, multiforme erythema, and diseases with no pathologic characteristics of vasculitis. The control group included 40 patients with other MTB-unrelated skin diseases, including stasis dermatitis, dermatitis, and eczema (Fig. 1). Their demographic and clinical characteristics are summarized in Table 2.

**Table 2:** The demographic and clinical characteristics of subjects.

	EN	NV	OV	Control
	n = 36	n = 27	n = 44	n = 40
Age Median (range)	39 (13–74)	40 (17–77)	38 (11–73)	48 (19–69)
Gender Male	5	4	9	9
Female	31	23	35	31
Disease duration Median (range)	1 y (2 w-10 y)	5 m (4 d-7 y)	2 y (3 d-12 y)	4 m (10 d-7 y)
Age at onset	19	14	19	10
Fever	7	4	3	0
Joint pain	4	3	6	2
History of TB	2	2	0	0
History of exposure to TB patients	0	0	3	2
BCG immunization^a^ (%)	83%	96%	100%	90%
Cases with QuantiFERON	32	26	38	40
Cases with tissue samples	23	19	34	40

a Recorded vaccination history or skin scars on the arm or pelma.

### Real-Time PCR Characterization of MTB in the Skin Tissue Lesions

To generate standard genes for quantitatively analysis of MTB, we amplified the *mpt64* and IS6110 gene fragments of MTB by PCR and cloned into pUC18 to generate a new plasmid that contained one copy of the *mpt64*, linker, and IS6110 genes, followed by DNA sequencing. After serious dilutions of the plasmid, we detected 1×10^3^–1×10^11^ copies/ml of the IS6110 gene and 1×10^3^–1×10^9^ copies/ml of the *mpt64* gene by Real-Time PCR, regardless of the presence or absence of human genomic DNA in our experimental conditions.

To determine the presence of MTB in the skin lesions, total DNA extracted from 117 skin lesion samples (23 EN, 19 NV, 34 OV, and 41 controls) and 115 out of 117 samples were subjected to PCR analysis of MTB. Real-Time PCR revealed that 7 out of 23 (30.4%) EN and 7 out of 18 (38.9%) NV samples were positive for the IS6110 DNA, respectively, which were significantly higher than 3/40 (7.5%) in the controls and 3/34 (8.8%) in the OV group (p<0.05). Similarly, nested Real-Time PCR assay indicated that no single IS6110-negative sample became positive for the *mpt64* gene. Second, 6/7 (86%) of the IS6110-positive EN samples were *mpt64*-positive and all IS6110-positive NV and control samples were positive for the *mpt64* gene. However, only 1/3 of OV samples were positive for the *mpt64* gene. Finally, there was no significant difference in the number of MTB copies among these groups of samples. Together, these data suggest that MTB infection may be associated with the development of EN and NV in some Chinese patients.

### MTB Antigen-specific IFN-γ Responses in the different Groups of Patients

To further determine the role of MTB infection in the pathogenesis of vasculitis, the MTB antigen-specific IFN-γ responses in 126 patients were determined by QuantiFERON assays. Following stimulation of peripheral blood mononuclear cells with MTB antigens *in vitro*, 124 out of 126 samples were valid and the other two samples were undetermined. As shown in Table 3, 19 out of 32 (59.4%) EN patients, 20 out of 26 (76.9%) NV patients, and 17 out of 36 (47.2%) OV patients displayed positive MTB antigen-specific IFN-γ responses. In contrast, only 6 out of 40 (15%) of the controls presented positive MTB antigen-specific IFN-γ responses. Apparently, the positive rates of MTB antigen-specific IFN-γ responses in the EN and NV patients were significantly higher than that of the controls.

**Table 3:** Characterization of IFN-γ responses in patients and MTB in the lesions by QuantiFERON and Real-Time PCR.

	EN	NV	OV	Control
	n = 36	n = 27	n = 44	n = 40
Cases with QuantiFERON, n	32	26	36	40
Positive	19 (59.4%**)	20 (76.9%**)	17 (**47.2%** *)	6 (15%)
PCR, n	23	18	34	40
Positive	7 (**30.4%** *)	7 (**38.9%** *)	3 (8.8%)	3 (7.5%)

* p<0.05, **p<0.001 vs. the controls.

### Concordance Analysis between Real-time PCR and QuantiFERON

There were 19 EN, 17 NV, 26 OV, and 39 control patients with valid results from the QuantiFERON and PCR tests. We found that the results from the QuantiFERON and PCR assays had no significant concordance in any group (Kappa value ranged from −0.152 to 0.17) or in all groups (κ = 0.137). Furthermore, the results from these two assays had a low agreement of 37% and 35% in the EN and NV groups, respectively, but had a high agreement of 62% and 85% in the OV and the control groups, respectively. There was a statistically significant difference among them (p<0.01). In addition, the QuantiFERON assay had a higher positive rate than that of the PCR assay. Although the difference in the positive rate between two assays was not significant in the EN or NV group (p = 0.146 and 0.065), it was statistically significant in all groups of patients (p<0.001).

## Discussion

There was little concordance between these two assays for detecting TB infection in our study. The lack of concordance may be not surprising because these two assays detect different targets by different methodologies. While the QuantiFERON detects immune response to MTB-specific antigens in individuals with latent TB infection, the real-time PCR assay detects the bacterium-specific genomic sequences, a measure of TB infection. Given that MTB-specific T cell immunity can control bacterial replication and infection it may be reasonable to detect potent QuantiFERON responses, but negative PCR detection and verse visa in immunocompromised individuals. Indeed, these methods for detecting MTB infection are not well concordant in active TB patients. Usually, MTB is mainly detected some patients with active TB, but rarely in individuals with latent TB infection. This is why culturing or PCR for detecting MTB should be performed several times on different specimens. It is well known that the rate of detecting MTB by PCR is dependent on the type of specimen, bacteria loads and threshold of the PCR method. In our study, there were limited lesion tissues usually from one location, which were hard for several PCR tests. In addition, the immune function of individual patients may affect the detection of antigen-specific T cell immunity. Finally, there might be some false positivity and negativity in both assays, which may also contribute to the lack of concordance. Although there was no concordance, these two assays were very effective in detecting TB infection immunologically and bacteriologically, respectively.

Currently, real-time PCR tests were normally used to detect MTB with a high specificity and sensitivity. A previous study has reported that the sensitivity and specificity of PCR for detecting MTB in stools samples were 100% and 97.3%, respectively, and the PPV was 88.9% and the NPV 100% [26]. A similar nested real-time PCR targeting the *mpt64* revealed a high sensitivity (95.8%) and specificity (100%) for 24 clinically suspected TBM patients, respectively, and the PPV and NPV were 100% and 97.2%, respectively [27]. Due to the lack of gold standard, the performance of QuantiFERON-TB Gold in detecting latent TB is hard to evaluate. In evaluating active TB, a meta-analysis concluded the pooled sensitivity was 76% (95% CI, 72% to 80%) for 22 QuantiFERON-TB Gold studies, and the pooled specificity was 98% (CI, 96% to 99%) for 16 QuantiFERON-TB Gold studies [21].

The IS6110 is an insertion sequence, which specifically and widely exists in *Mycobacterium* strains. The presence of the IS6110 gene in the EN and NV lesions remains controversial. While Baselga et al [9] reported the presence of the IS6110 gene in 77% of the paraffin-embedded NV tissues, others failed to detect the presence of the IS6110 in the EN and NV tissues [28]. We detected the IS6110 gene in 38.9% or 30.4% of the fresh NV and EN tissues. The positive rates for detecting the IS6110 gene in the lesions from our study were lower than that of Baselga’s research (77%). However, our findings were in disagreement with those of others [28] and suggest that MTB infection is associated with the development of EN and NV in some Chinese patients. The difference may stem from different genetic populations, MTB epidemic severity, and varying efficacies in extraction of DNA from the lesions and PCR amplification. Given that the paraffin wax interferes with the efficacy of DNA extraction and PCR amplification, we isolated DNA from fresh lesion tissues for PCR of the IS6110 gene, which may increase the efficacy of PCR and detection rate of MTB in tissue lesions.

To further confirm the existence of MTB DNA, we performed nested Real-Time PCR to detect the *mpt64* gene, which is a conservative gene for the MPT64 in a minority of MTB [26], [27], [29]. We detected the *mpt64* in most IS6110-positive tissues, except for three tissues, in which high copies of the IS6110 were detected. We speculate that these patients may have non-tuberculosis (NTB) mycobacterial infection. The presence of both the IS6110 and *mpt64* in the EN and NV lesions clearly indicates that MTB infection may contribute to the pathogenesis of EN and NV in some Chinese patients.

MTB infection usually induces Th1 responses, which secrete IFN-γ and can be detected by QuantiFERON assay [30]–[32]. We detected that the positive MTB-specific IFN-γ responses in EN and NV patients was similar to that of active TB patients [21]. The high positivity may include some patients with latent MTB infection. Indeed, 15% of the control patients displayed positive MTB-specific IFN-γ responses, consistent with a previous study [33], and supported the notion that Chinese have a high rate of MTB infection. Therefore, the results of the MTB-specific IFN-γ responses should be carefully interpreted. Simultaneous detection of MTB genes and MTB-specific immune responses should be valuable in determining the etiology of EN and NV in Chinese patients.

We found that the ratios of male patients with EN or NV to female patients were 1∶5 or 1∶6 and that gender variation was consistent with previous studies [1], [2], [12], [13]. Although endogenous female hormones may contribute to the development of EN and NV, the precise reasons causing the higher frequency of female patients with EN and NV are unclear. Interestingly, a previous study has shown that male patients are usually associated with active TB [34]. In our study, we found that positive rates of MTB detection in male and female patients with EN were 50% (2 of 4) and 26% (5 of 19), respectively, and that the positive rates of MTB detection in male and female patients with NV were 67% (2 of 3) and 31% (5 of 16), respectively. Clearly, the positive detection rates of MTB in male patients were higher than that in female patients, although there was no significant difference due to the small sample size. We are interested in further investigating whether MTB infection is more associated with the development of EN and NV in males or females in Chinese.

The pathogenesis of EI has been in debate. Although NV has been thought not to be tuberculous in origin, the definition between NV and EI of Bazin is vague. Our study revealed that some patients with either EI of Whitfield or Bazin were associated with MTB infection. It is possible that EI is a type of clinical manifestation of latent MTB infection. Histopathologically, most EI had diffuse septal lobular panniculitis with vasculitis in the small venules of the fat lobule [35]. A previous study has suggested that adipose tissue may be the reservoir of MTB, which is associated with either active or latent TB [36], linking the pathogen and the histopathology together. When the bacilli in the adipotypes become active and can be released, the free bacilli or large amount of antigens may mediate type III and type IV hypersensitivies, causing granuloma formation and primary vasculitis. In addition, the “foamy macrophages” also contribute to the pathogenesis of vasculitis. In most panniculitis, the development of vasculitis in the fat lobule can recruit neutrophils, lymphocytes, and a large number of macrophages, which can engulf the necrotic adipocytes and form characteristic “foam cells”. A recent study also demonstrates that oxygenated mycolic acids from MTB also play a crucial role in the differentiation of macrophages into “foam cells”, which may constitute a reservoir of tubercle bacillus for its long-term presence within its human host [37]. Although some NV patients had pulmonary TB, most patients did not have typical TB symptoms other than tuberculids. Our study demonstrated that most NV patients had MTB infection, but were negative for MTB detection. Some patients may turn into active TB later. NV (or EI) may be a type of clinical symptom of latent MTB infection and that those patients may have been at non-symptom stage of MTB infection for a while. If genuine, NV may provide a relevant clinical model for the study of latent TB and persistent non-replicating mycobacteria.

One patient with a 2-year history of NV was diagnosed with lymph node TB. Her QuantiFERON test was strongly positive, but her MTB DNA detection in her lesions was negative. She received formal anti-MTB treatment, and she had no relapse of lower limb skin rashes during the one year follow-up. Another two EN patients (one male and one female) were positive for both QuantiFERON and real-time PCR tests, but were negative for chest X-rays. They were treated with anti-MTB chemotherapy, and their lesions were cured without relapse during the one-year follow-up. Currently, patients with EN or NV are usually treated with glucocorticoids, which are not preferable for patients with TB. Therapeutic treatment with anti-TB medicines may be important for the control of EN and NV progression. Indeed, anti-TB treatment has been suggested for EN and NV patients with positive TST. Long term anti-TB medicines may impair the liver function, and a positive TST does not distinguish BCG vaccination from MTB infection. Accordingly, we recommend that treatment with anti-TB medicines may be valuable in the management of EN and NV patients with both positive detection of MTB genes and antigen-specific IFN-γ responses.

### Conclusion

In this study, we found that the positive rates of the IS6110 and *mpt64* genes of MTB in the EN and NV lesion tissues and MTB-specific IFN-γ responses in the EN and NV patients were significantly higher than that of the controls. These novel data suggest that MTB infection may contribute to the pathogenesis of EN and NV in some Chinese patients. Therefore, simultaneous detection of MTB infection-related microbiological and immunological evidence may help in the management of patients with EN and NV. We recognized that our findings were based on one center and that further studies from multiple centers in a bigger population are warranted to validate the findings.
